# A qualitative evaluation of the impact of a palliative care course on preregistration nursing students’ practice in Cameroon

**DOI:** 10.1186/s12904-016-0106-7

**Published:** 2016-03-31

**Authors:** Nahyeni Bassah, Karen Cox, Jane Seymour

**Affiliations:** School of Health Sciences, University of Nottingham, Queen’s Medical Centre, Nottingham, UK

**Keywords:** Transfer of learning to practice, Palliative care, Nursing education, Preregistration nursing, Resource-poor countries

## Abstract

**Background:**

Current evidence suggests that palliative care education can improve preregistration nursing students’ competencies in palliative care. However, it is not known whether these competencies are translated into students’ practice in the care of patients who are approaching the end of life. This paper seeks to contribute to the palliative care evidence base by examining how nursing students in receipt of education report transfer of learning to practice, and what the barriers and facilitators may be, in a resource-poor country.

**Methods:**

We utilised focus groups and individual critical incident interviews to explore nursing students’ palliative care learning transfer. Three focus groups, consisting of 23 participants and 10 individual critical incident interviews were conducted with preregistration nursing student who had attended a palliative care course in Cameroon and had experience caring for a patient approaching the end of life. Data was analysed thematically, using the framework approach.

**Results:**

The results suggest that nursing students in receipt of palliative care education can transfer their learning to practice. Students reported recognizing patients with palliative care needs, providing patients with physical, psychosocial and spiritual support and communicating patient information to the wider care team. They did however perceive some barriers to this transfer which were either related to themselves, qualified nurses, the practice setting or family caregivers and patients.

**Conclusion:**

The findings from this study suggest that nursing student in receipt of palliative care education can use their learning in practice to provide care to patients and their families approaching the end of life. Nevertheless, these findings need to be treated with some caution given the self-reported nature of the data. Demonstrating the link between preregistration palliative care education and patient care is vital to ensuring that newly acquired knowledge and skills are translated and embedded into clinical practice. This study also has implications for advocating for palliative care policies and adequately preparing clinical placement sites for students’ learning and transfer of learning.

## Background

Palliative care education is gradually being incorporated into preregistration nurse training programs, particularly in resource-rich countries [1–5]. This is important to ensure that generalist nurses can respond to increasing needs for palliative and end of life care, and use the palliative approach in their everyday encounter with patients approaching the end of life [2, 6–8]. Some studies reporting the development, implementation, and evaluation of the impact of palliative care education with preregistration nursing students have been published. A modified systematic review [1] and an integrative review [2] of this evidence suggests that palliative care education has a positive impact on nursing students’ attitudes towards care of the dying patient and, to some extent, on their palliative care knowledge and self-perceived competence in palliative care. Although this is encouraging, it is still not known whether these competencies are eventually translated into behaviors that can lead to improvements in patient and family experiences of care, and care outcomes. This paper seeks to contribute to evidence about the education of preregistration nursing students in palliative care by examining how students in receipt of palliative care education in Cameroon report transfer of learning to practice, and what they perceive as the barriers and facilitators of this learning transfer, in a resource-poor context.

**Fig. 1:**
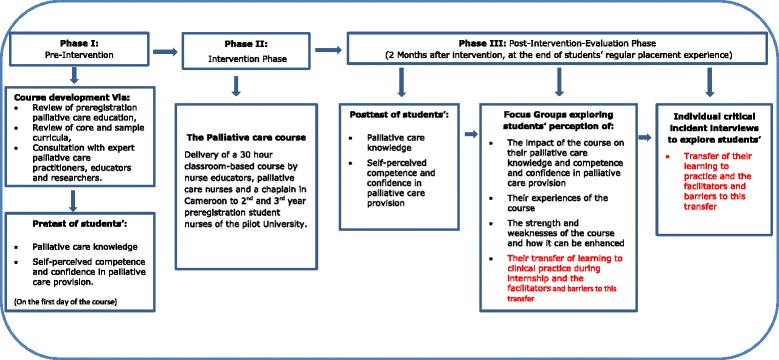
Diagrammatic presentation of the quasi-experimental study design

The need for palliative care in resource-poor countries is enormous, yet its provision is significantly limited [9–11]. In Cameroon, as in other resource poor countries, there are few dedicated palliative care services, palliative care drugs are not found on the national drug list, and morphine is highly restricted. Moreover a policy framework for palliative care is lacking from the National Health Strategic plan and palliative care is not a regular feature of health professional training in Cameroon [12]. In the context of these issues, dying from cancer, HIV/AIDS, and other non-communicable chronic diseases in Cameroon is often significantly associated with pain, poor control of other symptoms and lack of psychosocial support [13]. Thus Cameroon is an example of a setting in which it is urgent to consider how to address gaps in the WHO public health model for palliative care [14], namely: policy, education, implementation and drug availability. We focus on the provision of palliative care education to preregistration nursing student since work-force preparation is vital to the development of palliative care policies as well as to care implementation and drug availability. [15]. The paper draws on one aspect of longitudinal quasi-experimental single group pretest-posttest study, that employed both quantitative and qualitative methods of data collection and analysis to develop, pilot and evaluate a 30 h course in palliative care for second and third year nursing students (Fig. 1).

Registered nurses are the mainstay of the care of the dying, and as such should be competent and confident in palliative care [16]. Yet there is evidence of the lack of palliative care competencies among registered nurses and nursing students, internationally [1, 17, 18]. In Cameroon, some palliative care seminars for registered nurses have been delivered through partnership between local institutions and international collaborators and some nurses have travelled to other countries to undertake a palliative care specialization course [13, 19]. However, these educational approaches are expensive and not accessible to every nurse in Cameroon. Davies and Higginson (2004) [20] have argued that it is unrealistic to address the scale of needs for palliative care by expanding the specialist workforce; improving the education of healthcare professionals at the undergraduate level is potentially a more feasible strategy [6]. With this in mind, a palliative care course was developed for preregistration nursing students in Cameroon.

### The palliative care course

The palliative care course was a 30 h classroom based course underpinned by the experiential learning theory. The course was developed via: a modified systematic review of the literature [1], review of core curricula developed by international organizations [21–23], consultations with palliative care nurses and nurse educators in Cameroon, and consultation with expert palliative care educators and researchers in the UK.

The course was inserted into the regular nurse training program of one University in Cameroon, and piloted with volunteer second and third year nursing students, during the second semester of the 2013/2014 academic year. It was delivered in 5 days, spaced over one month. A major strategy in this study was to use local and affordable resources, to aid future sustainability, if successful. In this light, the course was delivered by 3 nurse educators (including the lead author), 2 specialist palliative care nurses and a chaplain in Cameroon. These course facilitators were selected based on their academic and professional backgrounds as well as their prior teaching experiences on the topic (s) they facilitated (Table 1). Educational resources and supportive materials were provided to the facilitators and students to enhance teaching and learning during the course. Each facilitator received a copy of the palliative care toolkit by Lavy and Woodridge (2008) [24] and its associated trainers’ manual [25], and were given some palliative care textbooks and manuals (Table 2) on the topics to be facilitated. Each participating nursing student received a copy of the palliative care toolkit, and also had access to palliative care text books, and manuals.

**Table 1:** Summary of palliative care course content and facilitators

Sessions	Content	Facilitator
Module I: Introduction to the principles and practice of palliative and end of life care		
Session 1	Palliative Care I (Concepts, meanings and principles, the need for Palliative care, international developments in palliative care	Lead author and a palliative care nurse
Session 2	Palliative Care II (the need for palliative care in Cameroon, palliative care models, palliative nursing skills)	Lead author and a palliative care nurse
Module II: Communication skills and Breaking Bad News		
Session 1	Communication in palliative care	Nurse educator with communication skills training
Session 2	Breaking bad news	Lead author
Session 3	Basic principles of HIV/AIDS counselling	Palliative care nurse
Module III: Pain and other symptom management		
Session 1	Pain and pain assessment	Lead author
Session 2	Pain management I (pharmacologic)	Palliative care nurse
Session 3	Pain management II (non-pharmacologic)	Palliative care nurse
Session 4	Other symptom management I	Palliative care nurse
Session 5	Other symptom management II	Palliative care nurse
Module IV: Psychosocial, spiritual, and ethical issues in palliative and end of life care		
Session 1	Psychosocial, ethical and legal issues in palliative care	Nurse educator, with experience in teachings about ethical and legal issues in nursing
Session 2	Spiritual care	A chaplain
Module IV: Dying and death; loss, grief and bereavement management		
Session 1	Dying, dead and after dead care	Palliative care nurse
Session 2	Bereavement management	Chaplain
Session 3	The palliative care nurse in loss grief and bereavement management	Lead author

**Table 2:** Palliative care textbooks and manual available to course participants

• Becker, R. (2010) Fundamental Aspects of Palliative Care Nursing. 2nd ed. Cromwell Press: Trowbridge
• Kinghorn, S. and Gaines, S. (2007) Palliative Care Nursing: Improving End of life Care. Churchill Livingstone: Edinburg.
• Payne, S. Seymour, J. and Ingleton, C. (2008) Palliative care nursing: principles and evidence for practice. 2nd ed. McGraw-Hill: Open University Press.
• Mari Lloyd-Williams (2008) Psychosocial Issues in Palliative care. 2nd edn. New York: Oxford University Press,
• Qneschuk, Hagen and McDonalds (2012) Palliative Medicine: A case based manual. 3rd edn. Oxford: Oxford University Press
• Wittenberg, Goldsmith, Ferrell and Ragan (2013) Communication in Palliative Nursing
• Goldman, Hain and Liben (2012) Oxford textbook for palliative Care for children. Oxford: Oxford University Press
• Renzenbrik (2011) Caregiver stress and staff support in illness and bereavement. New York: Oxford University Press
• McSherry (2008) Making sense of Spirituality in nursing and healthcare practice
• Regnoid D and Regnard (2011) A guide to symptom relieve in palliative care 2nd edn. Oxford: Radcliffe Publishing
• Vadivelu, Urman and Hines (2011) Essentials of pain management. Springer.
• Knapp, Madden and Fouler-Kery (2012) Pediatric palliative Care: the global perspective. Springer.
• Peter Hudson and Shiela Payne (2009) Family Carers in Palliative care
• Manroe and Payne (2011) Death, Dying and social differences. 2nd Edn. New York: Oxford University Press.

Students started their regular nurse training placement the week following the completion of the palliative care course, either in the medical, surgical, paediatric, outpatient or the haemodialysis units of a regional hospital. They practised under the supervision of their regular placement mentors. It was anticipated that students would encounter patients with palliative care needs during their placements, raising questions about whether they could apply their learning to the care of these patients and their families.

### Aim

This paper aims to:

1. Describe Cameroonian preregistration nursing students’ report of the transfer of their palliative care learning to practice during placement.
2. Identify the facilitators and barriers to this transfer perceived by the students
3. Examine what claim can be made about the perceived impacts of palliative care education on preregistration nursing students’ practice during placement.

## Methods

### Study Design

This paper reports a component of a longitudinal quasi-experimental single group pretest-posttest study that employed both quantitative and qualitative methods of data collection and analysis. In the first phase of the study the palliative care course described above was developed. In the second phase, the course was delivered to second and third year nursing students. In the third phase, an evaluation of the impact of the course on students’ palliative care knowledge, self-perceived competence and confidence in palliative care and transfer of learning to practice was conducted using: a pretest/posttest survey, focus groups and individual critical incident interviews. This paper presents findings from phase 3 of the study that explored students’ transfer of their learning to practice and the facilitators and barriers to learning transfer that they reported (Fig. 1).

### Data collection

Focus groups and individual interviews were used to collect data for this study. The focus groups were primarily aimed to evaluate the palliative care course as a whole. However, a number of questions investigated students’ transfer of their learning to practice during placement (Table 3). It also helped in the identification of students who had experienced caring for someone approaching the end of life in practice.

**Table 3:** Focus Group and Individual Interview guide

Focus Group Guide
• Before participating in this course what did you know about palliative care?
• What where your expectations when you registered for the course?
• How did your experience of this course compare with your expectations?
• Is there anything else you would have loved to learn from this course?
• How did your participation in this course benefit you?
• How did you use your learning from this course in practice during placement?
• What do you think have been influential in enhancing your learning in this course
• What did not quite work well during the course?
• How do you think your experience of this course could be improved?
Critical Incident Individual Interview Guide
• Can you describe the events or circumstances that led to this incident?
• Can you describe the role you played in this incident?
• Can you describe others who were involved in the incident and the role they played?
• What was the patient outcome?
• What do you think facilitated the care you provided in this incident?
• What barrier (s) prevented you from providing palliative care in this incident?
• What did you find most challenging in providing care to patients who require palliative care.

The individual interviews explored this transfer in-depth and were conducted using the critical incident technique (Fig. 1). This technique, developed by Flanagan in 1954 is a set of procedures for collecting direct observations of human behavior in such a way as to facilitate their potential usefulness in solving practical problems. Critical incidents are considered any observable human activity that is sufficiently complete in itself to allow inferences and predictions to be made about the person performing the act. This technique has also been widely used in nursing research [26, 27]. Critical incidents in this study were any encounters students reported with patients with life-threatening conditions and their families. Students were asked to narrate any incidences they could remember and to describe where they encountered these patients, what they could do or could not do for the patients and the facilitators and barriers they encountered to palliative care provision.

An interview guide (Table 3) was employed to encourage students to report behavioural descriptions, and where necessary, probes and prompts were used to elicit more information from the students. These interviews were started 2 months after the course, as soon as students had completed their placement, to facilitate recall of their experiences with patients and their families who were approaching the end of life during placement [26, 28]. The focus groups and interviews were all conducted in classrooms on the University campus. The focus groups were moderated by the lead author with a research assistant as note taker and lasted between 46–69 min. The individual interviews were also conducted by the lead author and lasted for an average of 30 min. All the interviews and focus groups were tape recorded and transcribed verbatim.

### Participants

The study participants were volunteer second and third year nursing students of the pilot University whom we judged had relevant clinical practice experiences with patients in need of palliative care thus facilitating the use of the experiential learning strategy. Compared to fourth year students, the other target group that could have been considered, these students were on campus for appropriate periods of time and timetabling of the new course could be adequately managed.

The total number of nursing students in the second and third year class of the pilot University, who were potential participants of this study, was 83. Sixty-nine of these students voluntarily registered for and started the course, but only 64 completed all five sessions of the course (Table 1). Of the 64 students who completed the course 23 (35.9 %) took part in the focus groups, 7 (30.4 %) males and 16 (69.6 %) females. Twelve (52.2 %) of them were from the 2nd year class and 11(47.8 %) from third year. One third year student did not show up for the focus group. Ten (15.6 %), of the 64 students, participated in the individual interviews, 3 (30 %) males and 7 (70 %) females. Two (20 %) of these students were from the second year, while 8 (80 %) were from the third year.

### Sampling strategy

**Fig. 2:**
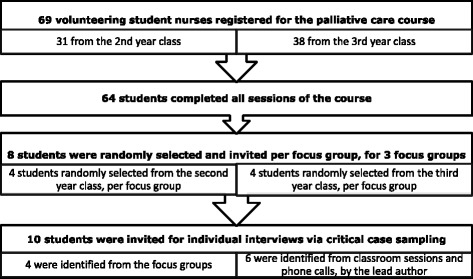
Sampling Procedure
- Second, a simple random sampling technique, via balloting of students’ course registration numbers [29], was employed to recruit the focus group participants. This strategy was to enhance recruitment of a representative sample, which could allow sufficient exploration and detection of consensus within and across the focus groups, and ultimately increase reliability of results [30, 31].
- Third, the critical case purposive sampling strategy [29] facilitated the recruitment of students for the individual critical incident interviews. This was conducted as follows:
  - ○ In the first place, students who mentioned during the focus groups that they came across patients with life threatening conditions were noted and later invited for individual interviews to explore this in-depth.
  - ○ In addition, the lead author visited the students in the classroom, and also made random phone calls to course participants, to inquire if they had experienced caring for patients with life-threatening/chronic conditions during placement. Students who attested to this were then invited for an individual interview, to explore the experience in-depth.

- First, the volunteer sampling strategy was used to recruit students for the course (Fig. 2).

### Data analysis

Preliminary thematic analysis of the focus groups enabled the recruitment of some students for the individual critical incident interviews. Data obtained from the individual critical incident interviews were read several times in order to become familiar with it. This process enabled the classification of the critical incidents and identification of critical interactions. The data were then categorised, as suggested by Flanagan (1954) [32], into location of experience and type of patient encountered, what they could or could not do for the patients and the facilitators and barriers they encountered to palliative care provision, and other useful characteristics.

In the second stage, a thematic analysis was conducted using the framework approach [33]. The data was coded and classified into main themes and subthemes, and used to develop a coding frame that was applied to the rest of the interviews. Although we developed and employed a thematic framework in coding the data [34], we were open to themes that emerged, based on the narratives of research participants. Thus constant examination, re-examination and comparison of interview transcripts was undertaken to identify any new themes and ensure rigorous analysis of students’ reported use of their palliative care learning in practice. The findings from the focus were integrated with that from the individual critical incident interviews to enhance data richness and depth of inquiry [35]. Qualitative interview transcripts were imported into the qualitative analysis software package, QSR Nvivo 10 [36], for data management, coding and analysis.

### Ethical considerations

Ethical approval to conduct this study was obtained from the University of Nottingham Medical School Ethics Committee and the University of Buea Institutional Review Board, prior to commencement. Prior to participation in this study, students were provided with an information sheet about the study and they voluntarily gave their consent to take part in the study.

## Results

The critical incident analysis yielded a total of 26 critical incidents, with an average of 2 incidents per student. Students reported encountering a wide range of patients with life-threatening and chronic conditions like HIV/AIDS, cancer, kidney disease, diabetes and hypertension, cardiovascular and respiratory diseases. Most of the patients encountered were adult or elderly patients. Some of these patients had no family members present.

Students’ transfer of their palliative care learning to practice with these patients is explained through 3 main themes: students’ reports of how they implemented their learning in practice, facilitators of palliative care learning transfer to practice, and barriers to the transfer of palliative care learning to practice (Table 4).

**Table 4:** Themes and subthemes

Themes	Subthemes
Students’ reports of how they implemented their learning in practice	1. Encountering patients and diagnosing the need for palliative care
	2. Communicating with patients, their families and the wider care team
	3. Assisting with physical care needs
	4. Providing psychosocial and spiritual care
	5. Reflecting
	6. Avoiding difficult interactions.
Facilitators of palliative care learning transfer to practice	1. The palliative care nurse
	2. Supportive nurses and family caregivers
Barriers to the transfer of palliative care learning to practice	1. Being a student
	2. Qualified nurses
	3. The practice setting
	4. Patients and family caregivers

### Students’ report of how they implemented their learning in practice

Students’ report of the transfer of their palliative care learning to practice in this study is described through six subthemes: encountering patients and diagnosing the need for palliative care, communicating with patients, their families and the wider care team, assisting with physical care needs, providing psychosocial and spiritual care, reflecting, and avoiding difficult interactions.

### Encountering patients and diagnosing the need for palliative care

Four main things were seen to initiate the encounter between nursing students and patients who required palliative care. Firstly, the students reported that, while on a general ward situation and carrying out ward routines with qualified nurses, they felt compassion and empathy towards patients, particularly when they perceived the patients had no family caregiver present, or were not adequately cared for by family caregivers or the wider care team:

“…when I saw her [an HIV/AIDS patient] lying helpless on the bed and without a family caregiver, although I was just passing around, I decided to talk with her… her look made me become empathic.” [Individual interview, Participant 1]

Secondly some students’ narratives show they encountered the patient when delegated responsibility for the patient’s care by qualified nurses. In responding to this obligation they identified some palliative care needs and thus offered certain palliative care interventions as described under the 5 subthemes that follow. However, they expressed some discomfort when delegated the care of someone they perceived was dying:

“She had HIV/AIDS ….[her] mother who was the carer [but] was kind of scared of her because of the changes that had occurred on her body. And there was no money to even buy the prescribed drugs and even the nurses in the hospital were like not paying attention to her. So she was lonely and kind of abandoned. When we were allocating patients to students one morning in the hospital, everybody [nursing student on internship] was like running away from her and unfortunately she fell on me” [Individual Interview, Participant 4].”

Thirdly, some students said that family caregivers conveyed information about patients’ behaviours which attracted their attention towards the patient. In other cases, the students were attracted to the patients based on the information the patients gave them during history taking. For example, one student said:

“The relatives were complaining to me that lately they have been talking to him and he does not respond and he spends the whole time sighing” [Individual interview, participant 10].

Fourthly, some students reported selecting certain patients because they saw it as an opportunity to apply their learning in practice:

“… She was HIV+ and was at the advanced stage. She was hospitalized there for more than three weeks… She also had other conditions… the bed sheets were wet and dirty. So when I saw it I thought it was time to apply the knowledge I acquired during the palliative care course. So the first thing I did was that I asked the nurse if she has had a bath, and the nurse told me no, that her daughter was supposed to do it, but she did not do it. So I planned to give her the bed bath, and I did so” [Individual interview, Participant 6].

During the above encounters, students often felt the need for palliative care when patients had the diagnosis of a life-threatening condition, when their level of dependency was significant or when they were seen as dying, by the students:

“She was HIV+, she was wasted, [her] condition was really getting worse every day. So I saw that palliative care will be really good for her” [Focus group, Participant 9].

### Communicating with patients and the wider care team

The students talked at length about how they communicated healthcare information with the patients, their families and the wider care team. They said they often seized any opportunity that arose to build rapport with patients. They also reported giving information to the wider care team that was relevant to patient care:

“…I learned that in palliative care we never say there is nothing we can do! When I sat and reflected on that, I asked myself what I can do for this patient. The only thing that was at my disposal was communication. I thought of active listening and how to communicate through talking. So that was what helped me. I went back to her, I paid attention to her, I was patient when she was talking, looking at her and trying to understand her” [Individual Interview, participant 4].

“Whenever there was a problem, I will always report to the nurses and even the doctor and share with them…and I was updating them on a daily basis…and representing the patient’s interest to them” [Individual Interview, participant 9].

A few students recounted responding to questions about life and death and breaking bad news to patients and their families. They expressed successes as well as difficulties in dealing with these difficult conversations:

“They constantly called me and ask whether there was hope for their father. To be candid, I told them the truth…I could say the truth firmly but with much softness” [Individual interview, Participant 5].

### Assisting patients with physical care needs

Students reported being engaged in delivering a wide range of active hands-on care, within the physical dimension of palliative care. Primarily, they described how they assisted patients who had life threatening conditions with their activities of daily living and comfort measures:

“I assisted her in performing her activities of daily living, with the hygiene I was there, to make up her bed, to help her with the sizth bath, and at times, I administered some medications” [Individual interview, Participant 2].

They also, although rarely, reported engagement in managing patients’ pain and other symptoms. Non-pharmacologic pain management measures were the major approaches employed by these students. This was possible due to the lack of prescribed pharmacologic pain management measures by the physician, requiring implementation by nurses. When pharmacologic measures were mentioned, these entailed the administration of prescribed analgesics, which were often mild analgesics or weak opioids. One student described an instance where she recognized the need for morphine in the management of a patient’s pain and communicated it to the physician, but said it was not considered:

“There was this patient, he was seriously in pain, and I asked the doctor, if there was nothing they could do about it, He said that no, he had been on so many analgesics that prescribing another one right now is not really a good idea. They wanted to prescribe Trabar [A narcotic analgesic, indicated for moderate to moderately severe pain], but I said she was really in pain… because we talked about that [during the palliative care course], the morphine, that when the pain was chronic, we had to start that one, but I don’t know” [Individual interview, Participant 5].

### Providing psychosocial and spiritual care

Students recounted providing psychological support and said they found this more straightforward to provide than other forms of palliative care support. They reported creating time, despite busy ward schedules, to be with patients with palliative care needs and their families in order to explore and listen to their concerns, and feelings. These students also reported teaching patients about their condition and treatment regimen, in order to help them to accept, adapt and live with it. They similarly reported that they educated family caregivers to enhance their ability to support patients and also to cope with their own distress:

“…when the young boy saw the father as we were cleaning (his wound) he started crying… after doing the cleaning, I had time to talk with him, to encourage him… like taking time to explain his father’s condition to him… considering his father’s health records, his condition was not changing, despite the medications that were being given. So with this, with the knowledge I had during the seminar [palliative care course], I knew that with this type of conditions, they can go home if they have a carer at home. So I asked the son if they have a close person at home, that is a nurse or someone who can offer nursing care…And then I also told him to collaborate with the nurses and that if he had any concerns, he can talk with the nurses and express his concerns” [Individual Interview, participant 10].

Moreover, the students said they often provided social support to patients who had no family caregivers with them in the hospital. One student narrated an incident where together with some of his classmates they advocated for the hospital’s social service department for support for a patient.

“…the patient was terminally ill with HIV…The patient had been in the hospital for three months and after two months, the relatives left that patient…we were concerned…We spoke with the patient… I consulted the charge nurse. The charge nurse said that is the problem they had been facing with this patient. But up to that time, they had not contacted the social service. So that is the proposal we gave. The charge nurse then went to the social service and the social service had to come in to call the patient’s family… the person came the following morning… So after that, we helped the patient to arrange his bed, we cleaned him and we handed the matter with the social service” [Individual interview, Participant 3].

Students also narrated incidences where they provided spiritual care, and this was often linked to formal religion. They reported praying, sharing bible stories and using religion oriented ways of reassuring and giving hope to patients and their families:

“…he said he thinks he wants to be in a Christian hospital, he wants to be where they will pray for him…So it was a bit difficult because the surgeon was absent so I went further and asked him if he needed any man of God to pray for him. He accepted and fortunately through one of our classmates, I asked her to call their pastor, and their pastor came and prayed for him. Immediately after, he could speak, and he got up on his bed and sat. And then after the pastor left, he went back to his bed and in less than one hour he died… I must say I actually saw an aspect of spirituality manifested” [Individual interview, Participant 3].

### Reflecting

Implementing palliative care in practice seems to have made students reflective. They were often either simultaneously engaged in reflections as they provided patients with palliative care or did so after administering care. There were instances where patients’ or family caregivers’ conditions made students to look inward in a search for meaning. In other instances, they reported acknowledging their reactions in the face of certain challenging interventions. These reflections seem important in helping students make sense of their practice with patients approaching the end of life, as well as making them think about the kind of care they might themselves receive at the end of life.

“…it actually struck me because if I were the one, lying on that bed, I don’t know whether somebody will do that to me…I think it’s really significant to me” [Individual interview, Participant 2]

### Avoiding difficult interactions

The students also talked about some ‘uncomfortable/difficult’ incidences that they avoided. Most particularly, they avoided talking about dying to patients and/or their families. It shows some students still experienced anxiety and lacked confidence in talking about dying, despite participation in the palliative care course:

“…but er, I spoke to her without mentioning death, death, death, or making her to know that she had an incurable disease or was in an advanced stage of the HIV/AIDS” [Individual interview, Participant 1].

### Facilitators of palliative care learning transfer to practice

#### The palliative care course

Students reported that the competencies acquired during the palliative care course were significant to their palliative care practice. Some students explained how the course helped them to provide palliative care by reflecting on the deficiencies of their pre-course practices with patients with life-threatening/chronic conditions:

“…I remember encountering so many incidences before we started the course but after the course, I realized that my approach was different, because I gained some knowledge” [Individual interviews, participant 3]

“Before the palliative care course, I had once worked in the intensive care unit…So when I did this course, I reflected back and I really saw that there were many things which I could have done at that time for them, but which I did not have that knowledge to do” [Focus group, Participant 15].

Some students cited incidences where particular interventions offered to patients were as a result of what they had learned from the course:

“…What was peculiar from the palliative care course that I learned is that; in palliative care we never say there is nothing we can do! When I sat and reflected on that, I asked myself what I can do for this patient. The only thing that was at my disposal was communication…”[Individual interview, Participant 4].

### Supportive nurses and family caregivers

A few students reported that the palliative nursing care provided by them was made possible by support of some qualified nurses. These were often related to activities of daily living and comfort measures:

“The nurses were encouraging me to go on with the procedure. They assisted me and they were telling me how to give the bed bath” [Individual interview, Participant 6].

Students also reported that when end of life patients and their families were supportive it was easier to provide palliative care:

“One of things that facilitated the care was the carers [family caregivers]. They were collaborative” [Individual interview, Participant 9].

### Barriers to the transfer of palliative care learning to practice

#### Being a student

Being a student emerged quite strongly as hindering the transfer of their learning from the palliative care course to practice. There were some incidences where students encountered patients with palliative care needs, but said they could not provide any palliative nursing interventions because of their limited remit of practice:

“…so my big challenge was the issue that I am a student, and it limited me a lot” [Focus group, Participant 20].

They also reported some incidences where they were not able to identify a palliative care need or decide what palliative care interventions were needed due to the lack of competence and confidence, as well as lack of experience:

“I came across somebody with a life-threatening condition, but I did not know how to really go about it” [Focus group, Participant 8].

#### Qualified nurses

Students reported that qualified nurses sometimes did not allow them the time they needed to provide palliative care. They reported being delegated other functions when they wanted to provide palliative care to patients whom they felt needed it. According to the students, these qualified nurses saw this as a waste of time, given that the patients were going to die after all:

“…they [nurses] told us that there are more important things to do and that there are patients that are there that can easily recover, we should take care of them, that that man has been there for 2 months and nothing is changing” [Focus group, Participant 20]

Students also reported that qualified nurses’ lack of knowledge about palliative care and negative attitudes towards care of the dying prevented the implementation of their palliative care learning, because they were not sure how to interact with these nurses to enable implementation of their learning in practice:

“…in addition to what [name of student withheld] is saying, what I also discovered is that the nurses in our setting know little or nothing about palliative care, so what I know and what they know actually differs. What I know about palliative care, they know little or nothing about that. So it’s as if I should also transfer the knowledge to them or teach them also, so that we can care for these patients” [Focus group, Participant 13]

### The practice setting

The students recounted how impractical it was to implement palliative care in a general ward situation given that there were often many patients in a ward in addition to those with life-threatening and chronic conditions. Thus the patients had diverse needs, and required diverse care. Moreover, the lack of a specialist palliative care team was another reported barrier:

“There are really barriers for example there is no particular setting where they place people with palliative care needs. So you have to come first of all and identify that this one is an incurable case, so the fact that there are no particular places for them, so there are no particular nurses or personnel allocated to them. So we find that we are dispersed to give care to everybody at the same time, so you can’t really concentrate on those dying patients because you have other patients in the ward you need to care for. So the fact that they do not have a particular place for them is really a challenge because you sometimes have the will to help them, but you don’t have the time to do it” [Individual interview, Participant 5]

In addition, they said that the existing hospital routines and the lack of a palliative care policy made it difficult to infuse palliative care into the existing practice. Moreover, the lack of resources for palliative care provision in the hospital and the unconducive physical environment where some patients who required palliative care were found were also mention as hindering their palliative care learning implementation in practice:

“…the hospital setting, especially where we go for practice, is already made in such a way that nurses have some fix things that they do, each morning, each afternoon, each evening or every time, they have a specific thing that they do… but it is not routine for you to see a nurse giving palliative care, even when the case is really advanced” [Focus group, Participant 16].

### Patients and family caregivers

Some students reported that it was difficult to provide palliative care when patients were difficult, expressing negative attitudes or when they were unconscious. Some student reasoned that patients were not ready or willing to engaging in discussions about dying and said that was a barrier:

“we went there to clean his wound and he was always looking sad, so I tried to ask him how he was feeling, he could actually talk just that he could not move, but he made a harsh sound that actually scared me, like he did not want to talk, he was not just willing to cooperate, so I found it very difficult to break the wall that he had built around him, so I ended up not doing anything” [Focus group, Participant 19].

They equally reported difficulty in providing palliative care when the family lacked palliative care knowledge and/or was death denying or giving up on the patient. This was a major issue because the family, rather than the patient were often in charge, and at times interfered with or disrupted intended care.

“…when I tried to discuss this with her sister, I saw that from her own idea of palliative care, she thought that palliative care was mainly for people that are dying, if somebody is going there it means the person is going to die. So for them they had a very big hope, they did not see their sister dying; they knew that she will recover and live normally. So for me, with this I had a problem implementing my knowledge” [Focus group, Participant 9].

Moreover, when the patients or their families lacked the needed care resources students’ practice was also hindered:

“Her drugs, palliative care also deals with drugs that she could not provide” [Individual interview, Participant 7]

## Discussion

This qualitative evaluation of the impact of a palliative care course on preregistration nursing students’ practice in Cameroon suggests that nursing student in receipt of palliative care education can use their learning in practice to provide care to patients and their families approaching the end of life. Students reported recognizing patients with palliative care needs, providing patients with physical, psychosocial and spiritual support and communicating patient information to the wider care team. There were, however, some barriers pertaining to the students themselves, qualified nurses, practice settings and to the patients and family caregivers, that hindered this learning transfer, as perceived by the students.

These findings need to be interpreted within the context of self-reported practice data. The use of retrospective critical incident interviews in this study could have resulted in distorted perceptions and sub-conscious editing of the incidents [26]. Moreover, students’ memory could be imprecise or some incidences might not have been reported [28]. Although some commentators have reasoned that researchers should be ‘confident in the ability of research participants to tell their story’ ([37], p. 306), studies in nursing have shown that direct observation of practice might yield more valid results [38, 39]. Direct observations can therefore be used to explore nursing students’ transfer of their palliative care learning to practice.

In addition, what is reported, at first sight, all sound like some ‘good nursing care’ which can be provided by any student nurse with a similar level of nurse training in Cameroon, irrespective of whether he/she took part in the palliative care course. It is also not clear whether and to what extent their behavior had changed after participating in the palliative care course, and how the observed behavior relates to the palliative care education received. We agree with other commentators that it can be quite challenging disentangling palliative care skills from routine nursing care skills in general practice settings [7], and measuring the direct or indirect impacts of education on practice [40].

However, a critical analysis of these students’ experiences portrays some key features of their reported practice, which could suggest they were using generalist palliative care competencies [7, 22, 23]. Some examples include being able to identify patients who needed palliative care, recognizing the need and advocating for morphine for a dying patient in severe pain, providing psychosocial support to family caregivers and educating them on patient care, and advocating for social support for a patient who had no family caregiver present. Moreover, some students reported that their learning from the palliative care course was a facilitator of the care they provided. In addition, these students, both in the focus group and individual interviews, self-evaluated their practices with dying patients, and said their post-course practices were comparatively better. Other studies have documented positive self-reports from nurses about the influence of a palliative care course on their practice, although these have involved qualified nurses rather than nursing students [41–43]. Therefore, although we cannot claim that the reported transfer to practice has come solely from students’ learning on the palliative care course, given the potential hidden value of these students’ nurse training course and of their interactions with patients, nurses and other members of the healthcare team it is a reasonable hypothesis to make. Further research of a different type would be needed to explore further; the only way to do this conclusively would be through a randomized controlled trial.

In reporting the implementation of their palliative care learning to practice, the students in this study recounted providing psychological support and said they found this more straightforward than other forms of palliative care support. This is an interesting finding that needs further examination in future research. Moreover, despite the palliative care course, the students still reported some avoidance behaviour and expressed a lack of competence and confidence to provide palliative care in some instances. Avoidance behaviour has been reported in other studies with preregistration nursing students [44, 45] and suggests the importance of not regarding palliative care education as a ‘one time’ endeavour. An effective model should incorporate plans for refreshers, support and mentorship post training and as students advance in their career as nurses [46, 47]. This is important particularly when students have not developed the confidence that can enhance their palliative care practice or when enthusiasm about and learning from the course may have waned.

Based on the findings from this study we can say that a number of barriers may prevent these students from transferring their palliative care learning to practice, and thus hinder the consolidation of learning and the learning of new skills. In this study, nursing students reported struggling with the lack of support from qualified nurses in the practice setting and lack of resources and policy for palliative care, while implementing their learning. They equally said it was challenging implement learning when the family was death denying or the patients were difficult. These resonate with previous findings from studies with practitioners and seem to confirm the claims that the integration of the palliative care philosophy in non-specialist palliative care settings can meet with resistance if staff attitudes, values and organizational frameworks are not clarified [17]. Some commentators have recommended educating health professionals, policy makers, and the general public about palliative care, and advocating for adequate palliative care policies and drugs as some ways of overcoming these barriers [48–50]. In view of these therefore, nurse educators need to: address the values that underpin care both at an individual and organizational level, educate placement sites and mentors about palliative care and how they can support students’ placement learning, lobby for palliative care policies with hospitals, as well as educate family caregivers and the general public to create their awareness and possibly gain their support for palliative care practice. These might to some degree improve on student nurses’ palliative care learning and learning implementation in clinical practice.

## Conclusion

Though it is important to demonstrate how palliative care education is effective in improving preregistration nursing students” palliative care competencies, it seems the greater measure of the effectiveness of this education is how students integrate these competencies in solving real problems with patients and their families who require palliative care. The findings from this study suggest that students can use their learning from a palliative care course in practice, although with some barriers. These barriers generally relate to the lack of awareness about palliative care amongst qualified nurses, other healthcare providers, patients and family caregivers, as well as to the lack of resources for palliative care in the health care system. Demonstrating the link between preregistration palliative care education and patient care outcome is vital to ensuring that newly acquired competencies are translated and embedded into clinical practice. This study also has implication for advocating for palliative care policies and adequately preparing clinical placement sites for students’ learning and transfer of learning. This can go a long way to improve on palliative care provision in resource-poor countries.
